# The Effectiveness of Brief Information and Self-Efficacy-Based Interventions in Influencing Snack Choices in Homeless Individuals

**DOI:** 10.3389/fpubh.2017.00293

**Published:** 2017-11-06

**Authors:** Chris Emmerson, Bev John, Susan Faulkner, Deborah Lancastle, Gareth Roderique-Davies

**Affiliations:** ^1^School of Psychology & Therapeutic Studies, University of South Wales, Pontypridd, United Kingdom

**Keywords:** homeless, nutrition, self-efficacy, snack choice, intervention

## Abstract

**Background:**

Homeless adults frequently experience poor nutrition. Research suggests raising self-efficacy and nutritional knowledge can increase healthy eating but that the choice of specific behavioral change techniques (BCTs) is also critical. This study investigated how BCTs, operationalized to increase nutrition knowledge and self-efficacy, might influence the choice of homeless adults when presented with a “healthy” and an “unhealthy” snack.

**Methods:**

A total of 125 homeless adults were randomly allocated to watch 1 of 4 brief films: “control,” “[nutrition] information only,” “self-efficacy” (aimed at increasing self-efficacy and general healthy eating knowledge), and “enhanced self-efficacy” (as “self-efficacy,” but the presenter identified themselves as a homeless adult). Post-film, participants were invited to choose between a healthy and an unhealthy snack. Data were analyzed using ANOVA and chi square.

**Results:**

Participants in the “enhanced self-efficacy” condition were significantly more likely to choose the healthy option compared to those in the control condition; for the “self-efficacy” condition, the difference was marginally significant. Perceived knowledge and self-efficacy were significantly associated and those with high self-efficacy were significantly more likely to choose a healthy snack regardless of condition.

**Conclusion:**

Homeless adults are more likely to make healthy snack choices if their nutritional self-efficacy is increased through encouragement by a peer.

## Introduction

Studies have consistently shown that homeless people have poorer health than the general population and that poor diet is a factor in this (1, 2). Homeless people face a range of obstacles to eating healthily, including a lack of money, food preparation facilities, and access to healthy food. These structural obstacles to eating a healthy diet may in turn lead to the development of unhealthy habits and preferences such as preferring fatty, sugary, or other densely calorific food for satiety, even on occasions when healthier alternatives are available (3).

Low fruit and vegetable intake (FVI) relative to guidelines and to the population as a whole has been identified as one dietary factor among homeless people that contributes to their relative ill health (4). Fruit, in both natural and processed forms (e.g., dried or as juice), can be provided easily as snacks in the context of services designed to support homeless people compared with, for example, healthy cooked meals or training on nutritional food preparation. Therefore, interventions that improve the uptake of these foods when offered within services have the potential to improve the dietary intake, and therefore, the health of homeless people in a relatively effective and cost-effective way.

Given the contexts and constraints on healthy eating interventions in homelessness services, development of a brief individual-level intervention related to fruit-based snacking was identified as the means of delivery and outcome most relevant to the aim of improving nutritional intake among homeless adults. Self-efficacy [“beliefs in one’s capabilities to organize and execute the courses of action required to produce given attainments”—(5), p. 2] and nutritional knowledge have been identified as constructs strongly related to FVI (6, 7). In addition, self-efficacy and nutritional knowledge also appear more amenable to delivery within brief interventions, when compared with approaches based around other theories identified within the systematic reviews, such as increasing social support or changing habits.

In relation to improving nutritional knowledge, there has historically been a consensus among researchers that knowledge-based models have failed to provide sufficient evidence that they meaningfully affect health behavior (8). However, this consensus has been challenged for basing assumptions on studies characterized by small sample sizes and poorly constructed and validated measurement scales designed for clinical populations (9, 10). Through developing and testing a number of interventions, this study offered an opportunity to consider the effectiveness of brief nutritional information-based interventions alone and in combination with approaches to increasing self-efficacy.

Studies evaluating health behavior generally and nutrition-related behavior specifically have been criticized for failing to accurately and consistently report the content of interventions, meaning the “active ingredients” causing change cannot be effectively identified, compared, or replicated (11, 12). A number of researchers have attempted to address this issue by defining valid and reliable taxonomies of behavioral change techniques (BCTs) that enable meaningful comparison and replication of interventions (13, 14). One meta-regression carried out on 53 healthy eating interventions found that interventions “prompting participants to self-monitor” were significantly more efficacious than interventions that did not use this technique. The efficacy of prompting self-monitoring was further increased if it was used with at least one other BCT of “prompt intention formation,” “prompt specific goal setting,” “provide feedback on performance,” or “prompt review of behavioral goals” (15).

The present study developed and tested brief interventions to influence the food choices of homeless people accessing accommodation and support services in Cardiff. Four interventions in the form of short videos were created. After watching the video to which they were randomly assigned, participants were offered a choice between a “healthy” snack (banana, carton of fruit juice, and packet of fruit and nut mix) and an “unhealthy” snack (packet of crisps, can of cola, and chocolate biscuit). The four videos featured residents of a local hostel reading scripts developed by the researcher directly to camera. The four scripts were developed to isolate and/or combine different aspects of healthy eating knowledge, approaches to self-efficacy development, and BCTs to distinguish between their effectiveness. It was hypothesized that the scripts including motivational elements derived from social cognitive theory [SCT, the broad theory within which the construct of self-efficacy is located (5)] would be more effective in encouraging their viewers to choose healthier snacks than control or “information only” scripts. It was further hypothesized that, in line with SCT, a script promoting identification with the presenter as a homeless person would be more effective in promoting healthy food choices than the video using only verbal persuasion.

## Materials and Methods

### Setting and Participants

Participants were homeless adults living in hostel accommodation or using day services for homeless people at six locations in Cardiff: the Huggard Day Centre, the Huggard hostel, Ty Tresillian hostel, Ty Gobaith hostel, YMCA Ambassador hostel, and YMCA The Walk, a support center attached to a hostel. “Homeless adults” was formally operationalized as those who were “homeless or vulnerably housed,” including those without any kind of accommodation of their own (e.g., street homeless) and those in shared accommodation provided specifically for homeless adults without security of tenure (e.g., residents of homeless hostels). Recruitment was carried out within services with the support of staff and managers and informed consent was gained from all participants.

### Procedure and Measures

Potential participants were approached on entering reception areas of the locations and informed that a study was being carried out about healthy eating and homelessness. The study procedures were briefly outlined and individuals were invited to take part. Those who agreed were escorted to a side room where informed consent was formally obtained. The researcher completed a brief questionnaire gathering basic sociodemographic data and details of recent food intake. In addition, participants were also asked to evaluate perceptions of their current health (“In general I would say my health is ….,” 4-point scale response), perceptions of nutritional knowledge (“Healthy eating is something I know a lot about”), the importance of good nutrition (“Healthy Eating is very important to me”), and current nutritional intake (“At the moment I am eating healthily”—all 5-point scales).

Finally, a 5-item nutritional self-efficacy questionnaire, developed and validated to a satisfactory level (Cronbach’s alpha = 0.87) (16) asked participants to assess their agreement on a 4-point scale to five phrases completing the stem sentence “I could eat more healthily and/or stick to eating healthy foods even if ….” The five completing phrases were: “I have to try several times until it works”; “I need a long time to develop the necessary routines”; “I have to rethink my entire way of nutrition”; “I do not receive a great deal of support when making my first attempts”; and “I have to make a detailed plan.” Summing responses to these questions gave a global nutritional self-efficacy score between 5 and 20.

### Interventions

Each participant was the shown one of four videos of a man speaking direct to camera. Two presenters were used, both recruited from a local hostel. The first video was a control in which the presenter described procedures for accessing emergency homelessness support. The second video (“information only”) presented information on the health benefits of a balanced diet, including the benefits of fruit and vegetable consumption. The third video (“self-efficacy”) not only included a summary of the information presented in the “information only” video but also included “verbal persuasion” of the value of eating a balanced diet and some specific advice on how to achieve this. In the final video (“enhanced self-efficacy”), the presenter identified themself as the resident of a homeless hostel and provided “vicarious experience” of their successes in eating more healthily.

All BCTs included were defined using the list developed by Abraham and Michie (13).

The “information only” condition included “provide information about behavior-health link” and “provide information on consequences.”

A summary of the BCTs used, with examples from the scripts, is presented in Table 1.

**Table 1:** Behavioral change techniques (BCTs) [numbered as Ref. (13)] applied in experimental manipulation videos, including derivation from theoretical frameworks.

Script	BCTs used	Example from script
Information only	(1) Provide information about behavior-health link (IMB) (2) Provide information on consequences (TRA, TPB, SCT, IMB)	“Foods such as bananas, carrots, dried fruit and fruit juice provide vitamins that are needed for everything from making new blood cells to keeping the immune system working well.”
Self-efficacy	(1) and (2) above plus (4) prompt intention formation (TRA, TPB, SCT, IMB), (5) prompt barrier identification (SCT), (6) provide general encouragement (SCT), (8) provide instruction (SCT)	“Deciding which positive changes matter to you and how you will overcome any difficulties can help you move from thinking about healthy eating, to believing you can do it, to action.”
		“It is not always easy to eat healthily if you are in a living in temporary housing like a hostel.”
		“Healthy eating is something you can do.”
		“With a little preparation and determination simple steps, like eating a piece of fruit every morning, can become part of your routine.”
Enhanced self-efficacy	(1), (2), (4)–(6), and (8) above plus (9) model or demonstrate the behavior (SCT)	“There are a few ways I have found to make it easier to move from thinking about healthy eating to believing you can do it, to making it happen. One is picturing the future and how healthy eating will bring the positive changes you want to happen.”

*IMB, information–motivation–behavioral skills model; TRA, theory of reasoned action; TPB, theory of planned behavior; SCT, social cognitive theory*.

The three videos including experimental manipulations all made reference to the relative nutritional value of the snack items subsequently provided to ensure participants were able to relate these to the concept of a healthy balanced diet and that the fruit-based snack offered following the video was therefore clearly identified as “healthy” and other snack as “unhealthy.” Nutritional information was developed with the support of an NHS community health dietician. All four videos were balanced for length (minimum 352 words, maximum 369) and linguistic complexity (range of Flesch Reading Ease scores = 62.4–66.9).

After viewing the video, participants were told that in return for taking part, they could choose one of two snacks, one consisting of a banana, carton of orange juice, and fruit and nut mix and one of a chocolate biscuit, can of cola, and packet of crisps. These “packaged” foods can be provided relatively simply and cheaply within homelessness services. The two snacks were placed on the desk in front of the participant in plastic boxes. Once the participant had chosen, they were debriefed and thanked for their time.

### Statistical Analysis

Participant characteristics were compared between groups using ANOVA or chi-squared tests for continuous and categorical variables, respectively, to check for baseline differences between groups. One-way ANOVA tests were also applied to compare responses between groups to the questions asking them to assess their health, knowledge of healthy eating, importance of healthy eating to them, and the healthiness of their current diet, as well as their global nutritional self-efficacy score. Note that the responses to these questions were treated as scales (1–5 for health and healthy eating questions, 5–20 for global nutritional self-efficacy). Difference between all four conditions is assessed with a 4 × 2 chi-square test. Each experimental condition was compared with the control condition using 2 × 2 chi-square test. Influence of baseline nutritional self-efficacy was also tested using a chi-square test to establish whether interventions worked at different levels in individuals with different initial self-efficacy. A three-way backward elimination log-linear analysis was performed on intervention, snack choice and (1) time since permanent accommodation (partitioned into quartiles) and (2) current accommodation to explore the possibility that different accommodation circumstances might affect the influence of each intervention.

## Results

Data from 125 participants were analyzed. Participant sociodemographic details by condition, with relevant tests for difference are given in Table 2.

**Table 2:** Sociodemographic characteristics of participants and test for differences between conditions.

	All participants	Control	Info	Self-efficacy	Enhanced self-efficacy	Test for difference	*p*
Mean age (SD)	36.11 (11.04)	35.88 (12.19)	36.71 (10.57)	37.50 (11.89)	34.47 (9.66)	*F* (3,121) = 0.42	0.736
**Gender**							
Male	109 (87%)	27	27	29	26	χ^2^ (3, *N* = 125) = 3.65	0.301
Female	16 (13%)	5	4	1	6
**Current accommodation**							
Hospital	1 (1%)	0	0	0	1	^a^	
Street	6 (5%)	1	1	3	1	^a^	
Sofa surf	5 (4%)	2	1	0	2	^a^	
EOS	29 (23%)	7	8	7	7	χ^2^ (3, *N* = 29) = 0.1	0.991
Hostel	75 (60%)	20	17	19	19	χ^2^ (3, *N* = 75) = 0.25	0.969
Shared house	6 (5%)	2	2	0	2	^a^	
TA flat	3 (2%)	0	2	1	0	^a^	
Permanent house^b^	11 (–)	3	3	5	0	^a^	
Mean months since settled (SD)	34.30 (44.88)	23.23 (29.31)	24.91 (30.21)	60.43 (64.96)	29.96 (38.47)	*F* (3,121) = 5.04	0.003
**GP registration**							
Yes	107 (86%)	27	27	25	28	χ^2^ (3, *N* = 125) = 0.31	0.957
No	18 (14%)	5	4	5	4		

*Percentages may not add up to 100 due to rounding*.

*^a^No statistical test available to test for difference due to low numbers*.

*^b^This category records participants who were permanently housed but were still using homelessness services following a period of homelessness. These participants are not included in figures for other rows in table. See Section “Materials and Methods” for further details*.

### Tests for Differences between Groups at Baseline

There were no significant differences between participants in different groups in terms of time of day, time since last ate, nature of last meal, location, or session date. Participants did differ significantly between conditions on “time since settled accommodation,” with those in the “self-efficacy” condition significantly more likely to have spent a longer time outside settled accommodation. However, there was no evidence that “time since settled” correlated with other variables and three-way backward elimination log-linear analysis suggested no interaction between this measure, intervention received, and snack choice (results not shown), suggesting that this variable did not systematically affect results. Additional tests were carried out to control for whether or not participants had eaten on that day, length of time since they had last eaten, the time of day, the location at which the intervention, and use of illicit drugs and alcohol. No significant differences were found between conditions (results not shown).

Table 3 reports means and SDs for all participants and across the four conditions with tests for responses to the four questions asking them to assess their health, knowledge of healthy eating, importance of healthy eating to them, and the healthiness of their current diet, as well as their global nutritional self-efficacy score.

**Table 3:** Means and SDs for all participants in response to perceived healthy, healthy eating, and nutritional self-efficacy questions and tests for difference between conditions.

	All participants	Control	Info	Self-efficacy	Enhanced self-efficacy	Test for difference	*p*
Perceived health	2.74 (1.1)	2.81 (1.06)	2.65 (1.17)	2.97 (1.03)	2.53 (1.14)	*F* (3,121) = 0.93	0.429
Knowledge of healthy eating	4.14 (1.03)	4.28 (0.81)	4.10 (0.98)	4.10 (1.32)	4.06 (1.01)	*F* (3,121) = 0.29	0.835
Importance of healthy eating	4.10 (1.26)	4.28 (1.14)	4.03 (1.47)	3.90 (1.37)	4.19 (1.03)	*F* (3,121) = 0.55	0.648
Healthiness of current diet	2.74 (1.54)	2.53 (1.61)	2.84 (1.64)	2.77 (1.59)	2.81 (1.36)	*F* (3,121) = 0.26	0.853
Nutritional self-efficacy	14.95 (3.77)	14.00 (3.92)	15.58 (3.39)	15.47 (3.87)	14.75 (3.84)	*F* (3,121) = 1.19	0.317

### Tests Comparing Intervention Groups

In total, 83 participants chose the “healthy” snack and 42 chose the “unhealthy” snack. Although the proportion of participants choosing the “healthy” snack followed the anticipated pattern, increasing with the assumed intensity of information and self-efficacy within each condition, there was not a significant difference between all four conditions. However, when experimental conditions were compared with the control condition, the “self-efficacy” condition approached significance (*p* = 0.059 compared with control) and the “enhanced self-efficacy” condition achieved significance (*p* = 0.019). The three results of these tests and the related odds ratios for choosing a healthy snack compared with the control condition are shown in Table 4.

**Table 4:** Results of 2 × 2 chi-square tests between control and other conditions.

2 × 2 Chi-square comparison between conditions	Chi-square result	*p*	Effect size (Cramer’s *V*)	Odds ratio (95% CI)
Control vs. information	χ^2^ (1, *N* = 63) = 1.36	0.244	N/A^a^	N/A^a^
Control vs. self-efficacy	χ^2^ (1, *N* = 62) = 3.55	0.059	0.24	2.75 (0.95–7.98)
Control vs. enhanced self-efficacy	χ^2^ (1, *N* = 64) = 5.5	0.0019	0.29	3.57 (1.2–10.6)

*^a^Effect size was only reported where p < 0.1, i.e., where the result could be considered to be approaching or achieving significance*.

A chi-square test showed that those scoring above the median on nutritional self-efficacy were significantly more likely to choose a healthy snack (*p* ≤ 0.001). However, there was no interaction between baseline nutritional self-efficacy, intervention and snack choice, with neither three-way nor two-way effects remained in the generating class of a three-way backward elimination log-linear analysis.

Neither three-way nor two-way effects remained in the generating class of models for the intervention, snack choice, and time since permanent accommodation or current accommodation.

## Discussion

A direct comparison of the control condition indicated that the “information only” intervention did not significantly affect participants’ snack choice. However, the difference in snack choice between those in the control condition and those receiving an intervention that added BCTs aimed at developing nutritional self-efficacy through encouragement, instruction, and the prompting of intention formation and barrier identification approached significance, while the further development of content and presentation to model behavior by a self-identified peer resulted in snack choice that was significantly different to that of the control condition. While a number of studies [e.g., Ref. (17–19)] have provided evidence of the impact of self-efficacy derived theory on improving nutritional behavior among those of low socioeconomic status, these results support more specifically Bandura’s (5) analysis of how increasing “authenticity” through prompting identification with a specific individual might increase the likelihood that health-related messages are acted on. This study further applies the development of self-efficacy to a target group of homeless adults, a population with distinct and critical social and economic problems.

This suggests that brief interventions to raise knowledge alone will not influence snack choice of homeless adults. Since, given that participants reported giving healthy eating a high priority (see Table 4), we might consider this audience “primed” for healthy eating information, the implication is that services should assume that simply providing information on healthy is likely to be ineffective.

The finding that the “enhanced self-efficacy” condition, but not the “self-efficacy” condition, was significantly more effective than the control condition in promoting healthy snack choice suggests that, for this social group, having nutritional self-efficacy messages delivered by their peers makes those messages particularly salient. In terms of the effectiveness of BCTs, these results further suggest that it was the combination of prompting intention formation, barrier identification and providing general encouragement (used in both “self-efficacy” and “enhanced self-efficacy” interventions) with modeling or demonstrating the behavior (in the “enhanced self-efficacy” intervention alone) that constituted the “active ingredients.” This finding further develops the evidence base described by Abraham and Michie (13) and Michie et al. (14) on effective combinations of BCTs in designing effective health interventions. In particular, these results evidence the value of ensure clear linkages between theory and BCTs.

There was a significant difference between snack choice among those with high baseline self-efficacy compared with those of low baseline self-efficacy, suggesting that the hypothesized link between self-efficacy and healthy eating behavior exists. However, analysis of baseline self-efficacy, snack choice, and intervention showed no significant interaction between these variables. In other words, there was no evidence that the self-efficacy interventions had a greater effect on those with low baseline self-efficacy rather than high baseline self-efficacy.

There were no interactions found between intervention, snack choice, and either the length of time individuals had been homeless or the nature of their current accommodation.

There are a number of methodological issues with this study. The questionnaire may have cued a focus on healthy eating behavior, which may have led to a general bias toward making healthy snack choices even in the control condition. The binary division of the snack choice into deliberately cued “healthy” and “unhealthy” conditions may not have reflected the wide range of options available in most food outlets, whether available to the general public or provided by homelessness services. More sophisticated designs may allow interactions between self-efficacy, information, and snack to be explored in more detail. A further limitation was the lack of post-intervention measurement of self-efficacy, meaning the inference that differences in snack choice was due to increased self-efficacy is assumed rather than measured directly.

Given the importance of good nutrition to health and the challenges homeless people face in establishing and maintaining good nutrition, these findings suggest that services working with homeless people could enhance the support they offer their service users by developing peer delivered self-efficacy based brief interventions. Homeless people typically fall short of achieving reference intakes of a wide variety of dietary measures (20) even when they are engaged with services intended to support them in improving their health status (4). That this study provides evidence for effectively addressing these nutritional issues is suggested not only by the results of statistical tests for effectiveness but also by the potential cost benefit ratios of this approach. This intervention could be delivered *via* peers recruited within homelessness services and given basic training in the relevant principles and techniques. Such a structure could allow for many interventions, formal or informal at a relatively low cost with considerable potential benefit. Given also that the results suggest self-efficacy-based interventions are equally effective for those at all points on the continuum of homelessness, and also equally effective for those at all levels of baseline self-efficacy, these interventions may be valuable for range of services working with a wide range of homeless people. Future studies should consider following up participants over time to evaluate longer term changes to self-efficacy and diet and establishing and evaluating pilot schemes to train a cohort of homeless individuals to deliver brief nutritional self-efficacy interventions within homelessness services.

## Ethics Statement

This study was carried out in accordance with the recommendations of “BPS Code of Human Research Ethics” with written informed consent from all participants. All subjects gave written informed consent in accordance with the Declaration of Helsinki. The protocol was approved by the “School of Psychology Ethics Panel, University of South Wales.”

## Author Contributions

CE and GR-D were involved in all phases of the research, including the design, data collection, analysis, and the writing of the manuscript. BJ, SF, and DL contributed to the interpretation of data, writing of the manuscript, and approving the final version. All authors are accountable for all aspects of the work in relation to accuracy and integrity.

## Conflict of Interest Statement

The authors declare that the research was conducted in the absence of any commercial or financial relationships that could be construed as a potential conflict of interest.
